# Palliative care in Germany from a public health perspective: qualitative expert interviews

**DOI:** 10.1186/1756-0500-2-116

**Published:** 2009-06-30

**Authors:** Mareike Behmann, Sara Lena Lückmann, Nils Schneider

**Affiliations:** 1 Hannover Medical School, Centre for Public Health, Institute of Epidemiology, Social Medicine and Health System Research, Carl-Neuberg-Str.1, D-30625 Hannover, Germany

## Abstract

**Background:**

Improving palliative care is a public health priority. However, little is known about the views of public health experts regarding the state of palliative care in Germany and the challenges facing it. The main aim of this pilot study was to gather information on the views of internationally experienced public health experts with regard to selected palliative care issues, with the focus on Germany, and to compare their views with those of specialist palliative care experts. Qualitative guided interviews were performed with ten experts (five from palliative care, five from public health). The interviews were analysed using qualitative content analysis.

**Findings:**

Older people and non-cancer patients were identified as target groups with a particular priority for palliative care. By contrast to the public health experts, the palliative care experts emphasized the need for rehabilitative measures for palliative patients and the possibilities of providing these. Significant barriers to the further establishment of palliative care were seen, amongst other things, in the powerful lobby groups and the federalism of the German health system.

**Conclusion:**

The findings suggest that from the experts' point of view (1) palliative care should focus on the needs of older people particularly in view of the demographic changes; (2) more attention should be paid to rehabilitative measures in palliative care; (3) rivalries among different stakeholders regarding their responsibilities and the allocation of financial resources have to be overcome in Germany.

## Background

This study was a pilot study within a larger research project aimed at developing public health targets for palliative care in Germany. The project had three phases: (1) a standardized survey covering 442 stakeholders from the German health care system, for example patient representatives and institutions involved in health care policy, in order to identify problems and improvement measures for palliative care; (2) qualitative interviews with ten international public health and palliative care experts; (3) based on the findings of phases 1 and 2, a modified Delphi study with sixteen national public health and palliative care experts, in order to develop public health targets for palliative care in Germany. The aims, designs and methods of the overall project have been published elsewhere [1]. This paper reports on the findings of the qualitative expert interviews in project phase 2.

## Aims

The aim of the study was to gather information on the attitudes and views of internationally experienced public health experts with regard to selected palliative care issues, with the focus on Germany, and to compare their attitudes and views with those of specialist palliative care experts. Furthermore, we wanted to learn more about the basic understanding of palliative care prevailing among public health experts. It was intended to make use of the results in developing the standardized instrument for phase 3 of the project.

## Methods

### Participants

The experts were selected on the basis of their internationally recognized competence in the field either of palliative care or of public health. Such competence was assumed on the basis of the following main criteria for inclusion: that the experts should be either board members of international scientific organizations in the field of palliative care or public health with a focus on Europe, or else editors or co-editors of scientific journals in the field of palliative care or public health. The selection of the experts was performed on the basis of literature and internet searches as well as the authors' personal experience and the advice of other experts within the authors' own institution.

In view of the pilot character of the study and its explorative approach, the total number of participants was predefined in the study protocol as N = 10 (5 experts each from palliative care and public health) [2,3]. In order to obtain this number, we had to contact 24 experts in all. These were sent a written request to participate, and where necessary were later reminded by telephone. 14 declined to take part or did not respond. The participants each received an allowance of €100.

The demographic data of the participants is shown in Table 1. Each expert was assigned a number: PH 1–5 are public health experts, PC 1–5 are palliative care experts.

**Table 1:** Demographic data of the participants

	**Public health experts**	**Palliative care experts**	**Both groups**
**Mean age**	55 years	48 years	52 years

**Female**	-	3	3

**Male**	5	2	7

**Main education:**			
Medicine	4	4	8
Psychology	-	1	1
Business administration	1	-	1
**Additional education:**			
Social science	1	1	2

**Religion:**			
Protestant	2	2	4
Catholic	-	1	1
Christian (not otherwise specified)	1	-	1
Agnostic	1	-	1
No religious affiliation	1	1	2
Not stated	-	1	1

**Professional status:**			
Chair holders Research fellows	5 -	3 2	8 2

**Location of practice:**			
Great Britain	-	3	3
Germany	2	1	3
Austria	1	1	2
USA	1	-	1
Sweden	1	-	1

### Instrument

For the interviews a semi-structured interview guide was used [see Additional file 1]. The questions were designed on the basis of the literature and of bibliometric and our own empirical studies [e.g. [4]]. The key topics were intensively discussed in the interdisciplinary study group from the point of view of their relevance to the aims of the overall project [1].

Firstly, the interview guide was developed in German and tested on two public health and two palliative care scientists for comprehensibility and appropriateness. After revision the interview guide was translated into English, and then translated back in order to test its comprehensibility, meaningfulness, appropriateness and survey equivalence [5]. Finally, a pre-test took place with two native English speakers, from the fields of palliative care and public health respectively, who were not involved in the final study. As a result of this pre-test a final revision of the English guide was undertaken.

### Data collection

The interviews were performed by telephone by two members of the research group, digitally recorded and fully transcribed. The interview language was English (N = 4) or German (N = 6), according to the preference of the participants. Interviews took place from April to July 2008 and lasted between 16 and 45 minutes (mean: 27 min.).

### Data Analysis

The transcripts were analysed according to the qualitative content analysis of Mayring [6]. Each interview was analysed sentence by sentence and coded line by line in order to reveal all the thought processes and meanings contained in the data. Every code was constantly compared and contrasted as well as grounded in data. Codes were conceptualized into related categories and there was an ongoing search for core variables in the text.

All transcripts were read and analysed independently by two members of the research team and subsequently discussed and collated. Atlas.ti software was used for coding, text searching and categorizing the views expressed. Quotations from the German speaking experts included in the following were translated from German into English and crosschecked.

## Results

**Figure 1:**
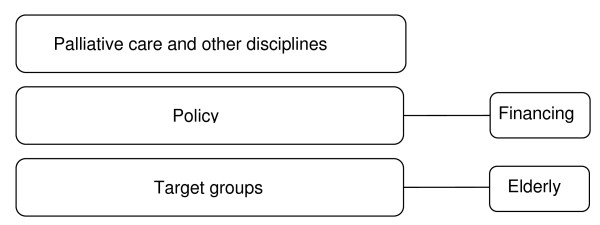
**Core categories**.

Three core categories were developed from the material (Figure 1). Table 2 shows the quantified data.

**Table 2:** Categories, topics and numbers of nominations

		**Nominations (N)**		
		
**Catagories**	**Topics**	**Public health experts**	**Palliative care experts**	**Both groups**
**Palliative care and other disciplines**	*palliative care suffers from a lack of acceptance by representatives of other disciplines*	-	2	2
	
	*the concepts of rehabilitation and palliative care are mutually exclusive*	4	-	4
	
	*need for rehabilitation for palliative care patients*	1	5	6

**Policy**	*inappropriate financing of inpatient palliative care services*	1	2	3
	
	*conflicts of interests between the different stakeholders*	1	2	3
	
	*development of palliative care is country and regionally specific*	3	5	8
	
	*more money required for palliative care*	3	4	7
	
	*allocation of money within the health care system*	1	1	2

**Target groups**	*palliative care for older patients*	2	5	7
	
	*increasing need for palliative care*	1	3	4

### Palliative care and other disciplines

When asked to define palliative care, the palliative care experts used terms and text passages that are familiar from the definition of palliative care provided by the World Health Organization and the European Association for Palliative Care more often than the public health experts did.

There was agreement between all the experts that palliative care is an interdisciplinary field with important intersections with other disciplines such as oncology, geriatrics, psycho-geriatrics, pneumonology, family medicine, cardiology and neurology. However, two of the palliative care experts believed that palliative care suffered from a lack of acceptance by representatives of other disciplines in Germany as well as in other countries:

*"(...) this is the lack, or the frequent lack, of acceptance among colleagues. (...), that the oncologists think they know what it [palliative care] is and don't understand that it has become an independent field. It is hard work to keep on trying to persuade them." *(PC 5; quotation translated from German).

The interfaces between palliative care and rehabilitation were extensively and controversially discussed. Four of the five public health experts were of the opinion that rehabilitation and palliative care do not overlap, but are two completely different areas of care that are mutually exclusive in terms of their fundamental concepts. For example, one of the public health experts stated:

*"By definition there are no overlaps between palliative care and rehabilitation, because rehabilitation implies health-related, occupational and social reintegration with a medium-term or long-term perspective, and these are different people from those who need palliative care." *(PH 2; quotation translated from German)

On the other hand, all the palliative care experts and one public health expert identified the need for rehabilitation for palliative care patients but thought it would be difficult to realize its delivery.

*"And of course we have a lot of overlaps with rehabilitation, we have similar issues. (...) Even in the neurological field there are many patients who are also undergoing rehabilitation, patients who have brain tumours (...)." *(PC 1; quotation translated from German)

### Policy

Both the public health experts and the palliative care experts emphasized the need for stronger political measures in order to further develop palliative care. In contrast to the recently improved financing of specialized outpatient palliative care in Germany, introduced with the recent health care reform in 2007, three experts criticized the still inadequate financing of inpatient palliative care services, e.g. palliative care units. Furthermore, there were calls for an overall political strategy to substantially improve palliative care, instead of isolated measures:

*"The most important precondition would be a better scheme of health care objectives and new legal provisions based on them. (...). I think we need a new concept for a better and well-rounded health policy. And it has to be supported by government policies." *(PH 5; quotation translated from German)

Three experts criticised the significant barriers to the realization of palliative care in Germany resulting from the numerous conflicts of interest between different stakeholders. In this context, one of the palliative care experts referred to the various powerful lobby groups and the federalism within the German health system:

*"At present, it is difficult to get heard by politicians. (...) in Germany everything is more difficult because of the complexity and size of the system. Before a bill can be passed, all the stakeholders have to be consulted." *(PC 4; quotation translated from German)

All experts pointed out that Germany could learn from improvement measures in other countries. The supportive and palliative care guidance of the National Institute for Clinical Excellence (NICE) in Great Britain was explicitly mentioned. Eight experts considered that the development of palliative care depended on country-specific frameworks. Furthermore, they believed that specific regional factors within countries played a major role. It was pointed out that there was a need for country-specific solutions:

*"But on the other hand I think that palliative care must take into account the specific conditions in each country and even within special regions, because palliative care in the city is not the same as in the countryside (...) so I think that the organization and setup of palliative care must differ between different countries, different societies." *(PH 1; original quotation)

### Financing

All experts, both from palliative care and public health, believed that funding problems were crucial barriers to the delivery of palliative care. Seven experts explicitly demanded the provision of increased funding for palliative care.

*"You have to have a system where you can build up these services financially, so you have to set aside resources for this." (*PH 1; original quotation)

It was stated that palliative care was in competition with other areas of health care for the available resources. The financing of palliative care has recently been improved in Germany. However, the allocation of money within the health care system was identified as a major problem by two experts:

*"And I think we put a lot of money into our health care system, but not necessarily into the right corners of it." *(PC 1; quotation translated from German)

*"I wouldn't want to regard it as being somehow natural that there should be a rich and a poor way of dying." *(PH 5; quotation translated from German)

### Target groups

In the context of demographic change, seven experts from both disciplines highlighted the relevance of palliative care for older patients, especially those suffering from polymorbidity and dementia; they believed a new understanding of palliative care should be developed.

*"Many diseases are defined in terms of norm values generated by young patients. We have to develop a new understanding of care for the elderly. If we apply approaches developed for a 30-year-old person to an 80-year-old person, we are declaring such elderly people to be sick and this is a misguided development. (...) We need health care derived from a better health concept." *(PH 2; quotation translated from German)

Four experts expected the need for palliative care to continue to increase in future, one of them drawing particular attention to non-cancer patients as a target group.

*"If we look at the demographic development, and indeed simply at the number of people who will be dying over the next 30–40 years, we can see that an enormous requirement is going to arise and that a lot is going to be expected of us in that respect." *(PC 1; quotation translated from German)

*"But increasingly, there's a recognition that specialist palliative care may be appropriate for patients who don't have cancer, but who have advanced or terminal disease." *(PC 2; original quotation)

## Discussion and conclusion

On many topics there was a wide measure of agreement as between the public health and the palliative care experts, but there were also relevant differences in their views, relating above all to the interfaces between palliative care and rehabilitation. Whereas all the palliative care experts were quite outspoken in their demand for rehabilitative approaches to be included in palliative care, four of the five public health experts were of the opinion that palliative care and rehabilitation are mutually exclusive. There may be a number of different explanations for these divergent views. It is conceivable that the public health experts would define palliative care more narrowly in terms of care for dying patients, so that rehabilitation is not, or is no longer, an option, whereas the palliative care experts also extend the palliative care concept to patients in the earlier stages of an incurable disease, when preventive and rehabilitative measures can often be very helpful in maintaining quality of life and functionality.

Another explanation might be that the public health experts understand rehabilitation more in terms of restoring the ability to work, as the interview quotation (PH 2) presented above seems to indicate. Finally, how far the perception of rehabilitative potential is influenced by the fact that the palliative care experts interviewed have closer practical relations with palliative patients than the public health experts do is a matter for debate.

Not surprisingly, in view of sociodemographic developments, older people and non-cancer patients were identified in our interviews as priority target groups for palliative care. Strengthening palliative care can significantly contribute to the quality of life of these patient groups [7-10]. In contrast to palliative care for older people, paediatric palliative care was not raised as a matter for discussion among our experts, which is somewhat surprising considering the importance of the topic [11]. This may suggest a lack of awareness. However, it is also possible that the focus on palliative care for adults and older people was reinforced by the nature of our central questions within the overall project [1].

The findings from the interviews suggest the existence of a variety of barriers to the implementation of palliative care in Germany and to its integration into the German health care system. Major problems were seen in the lack of clearly defined responsibilities as between health professionals and other stakeholders, and in the insufficient funding of palliative care services. Interdisciplinary rivalry and strong lobbyism are well known from other areas of the German health care system, and it seems that they are increasingly affecting palliative care now that it has become a new specialty with the need for appropriate funding by the statutory health insurance system [12].

With regard to the demand for more money for palliative care, it is notable that there was widespread agreement among palliative care and public health experts: the majority of the public health experts – who are not specialists in palliative care or involved in the delivery of services, and consequently are not to be suspected of any particular lobbying for palliative care – agreed with the palliative care specialists' opinion that palliative care needs to be better funded. There was some criticism of the distribution of financial resources in the German health care system: it was said that there was enough money in the system overall, but that it was not being properly allocated, so that funds were not being adequately applied to the care of very seriously ill and dying people. Above all, it was said, more money needed to be devoted to outpatient/non-residential care.

The recent German health care reform of 2007 promises significant improvements as it encourages specialist outpatient palliative care. For the first time, patients have a right to be given access to such specialist outpatient care. However, arrangements have not yet been determined for its practical realization [12,13]. It should be borne in mind that this 2007 health care reform strengthens only specialist outpatient palliative care, but not the wide area of primary palliative care (which is above all the responsibility of family doctors).

## Limitations

Some limitations to this study need to be mentioned. The study consists of a convenience sample with only a small number of participants. This is methodically justifiable within the qualitative explorative research approach [2,3], but it needs to be borne in mind that (as goes without saying) its results are not representative of the views of public health and palliative care experts. However, it was not our aim to obtain representative results, but to explore experts' opinions in order to be able to take them into consideration in developing the standardized instrument for phase 3 of our project.

In view of the small number of participants and the method of recruiting, some selection bias may have occurred. Furthermore it should be considered that not all interviewees were able to answer all the questions in detail, as some of them had no profound knowledge of specific aspects of palliative care in Germany. Where possible we provided further information during the interview process, e.g. regarding numbers of hospice beds and palliative care teams, legal issues and political developments in this field.

## Competing interests

The authors declare that they have no competing interests.

## Authors' contributions

MB and SLL conducted the interviews, analysed the material and helped to draft the manuscript. NSCH conceived the study, participated in the analyses and drafted the manuscript. All authors read and approved the final manuscript.

## Supplementary Material

Additional file 1 **Interview guide**. The interview guide that was used for the telephone interviews with the experts. Click here for file
