# Supplementary material for: Palliative care in Germany from a public health perspective: qualitative expert interviews
Source: BMC Res Notes. 2009 Jun 30;2:116. doi: 10.1186/1756-0500-2-116 (PMC2715412; doi:10.1186/1756-0500-2-116)
Supplement: Additional file 1 — Interview guide. The interview guide that was used for the telephone interviews with the experts. [file 1756-0500-2-116-S1.doc]

**Additional file 1**

**Interview guide**

Public Health Experts:

1. How long have you been engaged in Public Health work and what is your main focus within the field?
2. Do you have any points of contact* with topics within the area of palliative care. If yes, which ones?

*🡪 Instructions for the interviewer: if the expert does not bring out any concise arguments, the interviewer can ask him/her to specify the answer, e.g. would you please state some of these points more precisely?*

* This includes seminars, research projects, conferences, presentations and lectures.

Palliative Care Experts:

1. How long have you been engaged in palliative care work and what is your main focus within the field?
2. Do you have any points of contact with topics within the area of Public Health, if yes, which ones?

*🡪 Instructions for the interviewer: if the expert does not bring out any concise arguments, the interviewer can ask him/her to specify the answer, e.g. would you please state some of these points more precisely?*

Understanding:

a. How do you personally define Public Health?

b. How do you personally define palliative care?

c. Who do you consider to be a palliative patient?

d. In your opinion, are there any overlaps and/or similarities between palliative care and other branches of health care?

*🡪 Instructions for the interviewer: ask him about other health care services which have not been referred to in the response. For example, where do you see the position of palliative care within the fields of oncology, family medicine, geriatrics, nursing and rehabilitation services?*

Status and development of palliative care:

a. How would you assess the development of palliative care over the past few years?

b. What are the barriers for the implementation of palliative care?

*🡪 Instructions for the interviewer: if limitations are stated only on the European level, further specific questions may be asked, e.g. could you define these factors in the individual countries as well?*

c. What are the promoting factors for the implementation of palliative care?

*🡪 Instructions for the interviewer: if promoting factors are mentioned only on the European level, further specific questions may be asked, e.g. could you specify these factors in the individual countries as well?*

d. Are there any particular measures that have contributed to the improvement of palliative care?

e. Do you think that improvement measures from other countries can be implemented in Germany? Why or why not?

*🡪 Instructions for the interviewer: if the person answers in a short way, the interviewer can ask about reasons for this opinion, further details about the previously mentioned measures or concepts.*

f. In your opinion, which measures are required to sustainably improve palliative care in Germany?

*🡪 Instructions for the interviewer: if the expert does not specify reasons for the statement, further specific questions may be brought up, e.g. would you please specify which promoting and/or inhibiting factors highlight these suggestions*

Other important aspects

Are there any further aspects or arguments that we need to discuss? Do you have any general remarks or comments?
